# Mapping connections between complex post-traumatic stress disorder and psychotic-like experiences among adolescents: a Gaussian and Bayesian network study

**DOI:** 10.1017/S0033291725000169

**Published:** 2025-02-25

**Authors:** Tommaso B. Jannini, Valentina Socci, Adriano Schimmenti, Grazia Terrone, Lucia Sideli, Luis Alameda, Monica Aas, Giorgio Di Lorenzo, Cinzia Niolu, Rodolfo Rossi

**Affiliations:** 1 Department of Experimental Medicine, Tor Vergata University of Rome, Rome, Italy; 2 Department of Biotechnological and Applied Clinical Sciences, University of L’Aquila, L’Aquila, Italy; 3 Department of Human & Social Sciences, Kore University of Enna; 4 Department of History, Humanities and Society, Tor Vergata University of Rome, Rome, Italy; 5 Department of Human Science, LUMSA University, Rome, Italy; 6 Department of Psychosis Studies, Institute of Psychiatry, Psychology & Neuroscience, King’s College London, National Psychosis Unit, South London and Maudsley NHS Foundation Trust, London, United Kingdom; 7 Service of General Psychiatry, Treatment and Early Intervention in Psychosis Program, Lausanne University Hospital (CHUV), Lausanne, Switzerland; 8 Department of Psychiatry, Instituto de Investigación Sanitaria de Sevilla, IBiS, Hospital Universitario Virgen del Rocío, Universidad de Sevilla, Spain; 9 Social, Genetic and Developmental Psychiatry Centre, Institute of Psychiatry, Psychology and Neuroscience, King’s College London, London, United Kingdom; 10 Department of Psychosis Studies, Institute of Psychiatry, Psychology & Neuroscience, King’s College London, London, UK; 11 Department of Systems Medicine, Tor Vergata University of Rome, Rome, Italy; 12 IRCCS Fondazione Santa Lucia, Rome, Italy

**Keywords:** Bayesian network analysis, cPTSD, gender, psychosis, psychotic-like experiences, trauma

## Abstract

**Background:**

Complex post-traumatic stress disorder (cPTSD) is a newly recognized condition characterized by core PTSD symptoms and disturbances in self-organization (DSO) that has been associated with psychotic-like experiences (PLEs). This study employs two psychopathology network approaches to identify which post-traumatic symptoms are related to PLEs in a sample of late adolescents. We propose that cPTSD symptoms play a crucial role in explaining the co-occurrence of trauma and PLEs.

**Methods:**

A sample of 1010 late adolescents provided measures of post-traumatic symptomatology and PLEs. We estimated the Gaussian graphical network structure of PTSD/cPTSD symptoms and PLEs and assessed their bridge centrality indices. Bayesian network analysis was then used to estimate a directed acyclic graph (DAG). Gender was set as a moderator in both Gaussian and Bayesian models.

**Results:**

Results show that affect dysregulation, a cPTSD domain, presented the highest bridge connection with the PLE cluster. Bayesian network analysis identified a pathway going from cPTSD items of worthlessness and relational dysregulation, to PLE items of paranoia and social anxiety. Additionally, we found relevant gender differences in network connectivity, with females showing higher connectivity compared to males.

**Conclusions:**

Our findings highlight the central role of affect dysregulation and negative self-concept in linking cPTSD to PLE symptoms, with specific differences according to gender. These insights underscore the need for targeted, gender-sensitive approaches in the prevention and treatment of PLEs among adolescents, emphasizing early intervention and tailored treatment strategies.

## Introduction

Adolescence is a critical period of life in which young individuals may be exposed to different stressful factors, including societal and academic pressure, issues in identity formation, and troubled relationships (Daniunaite et al., 2021; Rossi, R. et al., 2023a). These life experiences might facilitate major traumatic events and enduring psychological distress and functional impairment in vulnerable individuals. Stress-related disorders and psychotic phenomena represent two key conditions for subsequent psychiatric morbidity.

### Complex post-traumatic stress disorder (cPTSD)

Complex post-traumatic stress disorder (cPTSD) is a recently introduced nosological entity featuring both core PTSD symptoms (i.e. re-experiencing, avoidance, and hyperarousal) and *disturbances of self-organization* (DSO) (i.e. negative self-concept, relational dysregulation, and affect dysregulation) (Maercker et al., 2022). cPTSD is thought to stem from complex traumatic experiences, that is, prolonged traumatic interpersonal events occurring mainly during childhood and adolescence. DSO, and affect dysregulation in particular, is associated with more severe post-traumatic symptomatology and with worse clinical outcomes compared to PTSD, including a higher prevalence and severity of psychotic-like experiences (PLEs) (Mason et al., 2023; Rossi, R. et al., 2023b; Rossi, Socci, et al., 2023), suicidality (Jannini et al., 2023), substance abuse (Jannini et al., 2024; Schimmenti, Billieux, Santoro, Casale, & Starcevic, 2022), and dissociation (Hamer, Bestel, & Mackelprang, 2024; Longo et al., 2019). Although DSO symptoms might be highly superimposable with core borderline personality disorder (BPD) features, both conditions deserve distinct classifications (Powers et al., 2022). To this end, it is worth noticing that in cPTSD, emotional dysregulation manifests as chronic emotional numbing and difficulty in self-calming, often tied to stable feelings of guilt and shame. Conversely, BPD is marked by emotional lability, intense anger, and a fragmented sense of self that fluctuates with interpersonal stress. Relational dysregulation in cPTSD typically involves avoidance and fear of closeness, while BPD is characterized by volatile relationships driven by fears of abandonment (Ford & Courtois, 2021). Besides long-established structural equation studies, recent network analyses further highlight these distinctions, emphasizing unique symptom dynamics and underlying vulnerabilities tied to trauma exposure (Owczarek et al., 2023).

### Psychotic-like experiences (PLEs)

PLEs are sub-clinical psychotic phenomena that include subtle cognitive or perceptual abnormalities, hallucinations, and bizarre ideation that occur in the general population in the absence of functional impairment (Hinterbuchinger & Mossaheb, 2021). PLEs are more common in childhood and early adolescence, with an estimated prevalence of 17% in school-aged children and 7.5% in teenagers, declining to around 5% in adults (Kelleher, Connor, et al., 2012; van Os, Linscott, Myin-Germeys, Delespaul, & Krabbendam, 2009). Although, by definition, PLEs do not affect functional capacity, they are an established risk factor for maladaptive behaviors (Kelleher, Keeley, et al., 2012; Mariano et al., 2021), obsessive-compulsive symptoms (Rössler et al., 2011), substance use and suicide (Cederlöf et al., 2017), and most importantly, for subsequent transition to a full-blown psychotic disorder (Dominguez, Wichers, Lieb, Wittchen, & van Os, 2011). Moreover, substantial evidence suggests that PLEs may represent a common comorbidity across a wide range of psychiatric disorders. These include major depressive disorder (Baryshnikov, Suvisaari, et al., 2018), generalized anxiety disorder (Varghese et al., 2011), obsessive-compulsive disorder (Mawn et al., 2020; Pacitti et al., 2022), attention-deficit/hyperactivity disorder (Hurtig et al., 2011; Jeon, Lee, Cha, & Kwon, 2023), eating disorders (Solmi, Melamed, Lewis, & Kirkbride, 2018), and autism spectrum disorders (Vaquerizo-Serrano, Salazar de Pablo, Singh, & Santosh, 2022). However, while qualitative studies in this area remain largely absent, a well-established causative relationship akin to that observed in trauma-related disorders has yet to be identified.

### Association between cPTSD and PLEs

**Figure 1.**
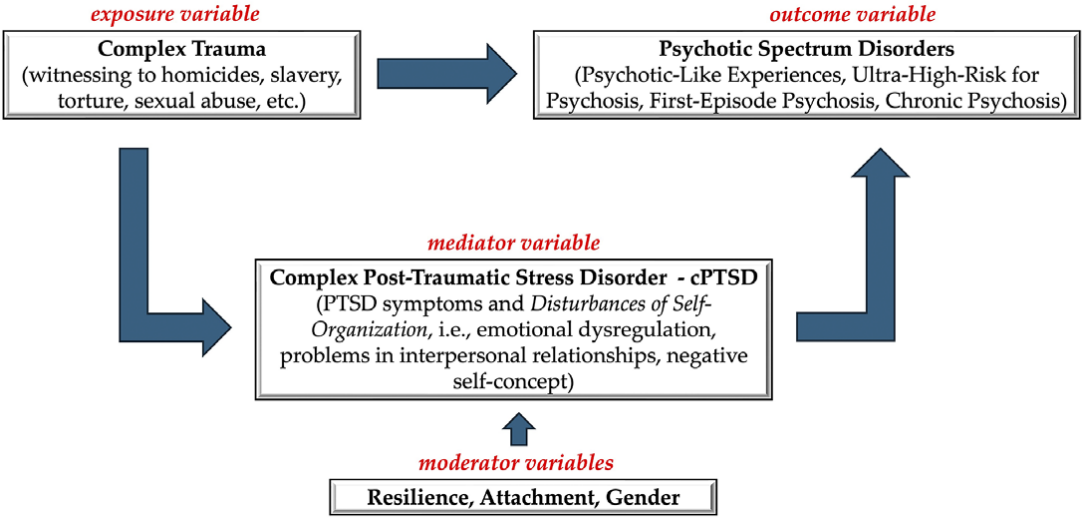
Theoretical framework of the complex trauma-psychosis relationship. Complex trauma might directly or indirectly, with complex post-traumatic stress disorder as a mediator, lead to psychotic-spectrum disorders. In the latter case, symptoms pertaining the *disturbances of self-organization* domain, that is emotional dysregulation, problems in interpersonal relationship, and negative self-concept are believed to act as primers of the onset of psychotic symptoms. Resilience, attachment, and gender have been reported as moderators of such associations.

Evidence suggesting an association between cPTSD and PLEs in adolescence has been growing recently (Figure 1). This connection is rooted in their shared relationship with complex trauma, a well-established risk factor for the whole psychotic spectrum, spanning subclinical manifestations (e.g. PLEs and ultra-high risk for psychosis) to overt clinical phenotypes (e.g. first-episode and chronic psychosis) (Alameda et al., 2020; Bloomfield et al., 2021; Campodonico, Varese, & Berry, 2022; Michel, Kindler, & Kaess, 2022; Sideli et al., 2020; Varese et al., 2012). Exposure to complex psychological trauma can disrupt normal information processing, leading to the formation of fragmented, emotionally intense memories that are poorly encoded with temporal, spatial, and sensory context—elements typically present in non-traumatic episodic memory formation. The resurfacing of these inadequately integrated memories may contribute to the development of trauma-related psychotic manifestations (Bloomfield et al., 2021; Onyeama et al., 2024).

Early studies have documented an increased prevalence of complex trauma in severe mental illness (Mauritz, Goossens, Draijer, & van Achterberg, 2013). More recently, research has extended this understanding, with an Italian study showing that cPTSD is associated with distressing PLEs in a non-clinical sample (Rossi, R. et al., 2023d). Similarly, another recent study identified a significant link between psychotic symptoms and cPTSD in a non-clinical population (Mason et al., 2023).

Models linking trauma and psychotic symptoms often conceptualize PTSD/cPTSD symptoms as mediators in the relationship between trauma and psychosis, a perspective supported by several reviews (Alameda et al., 2020; Sideli et al., 2020; Williams, Bucci, Berry, & Varese, 2018). Additionally, different studies have explored the role of several potential moderators of cPTSD-psychosis association, including resilience, attachment (Rossi, R. et al., 2023b; Rossi et al., 2021), and gender (Comacchio, Lasalvia, & Ruggeri, 2019), with women exposed to adverse childhood experiences displaying an earlier age of onset.

Nonetheless, existing evidence has primarily focused on individual aspects of cPTSD, with limited research exploring the condition as a unified syndrome that incorporates all its core components. Findings examining the full spectrum of cPTSD symptoms and their associations indeed remain limited (Longden et al., 2020; Mason et al., 2023; Panayi et al., 2022).

### Network analysis

Network models of psychopathology have gained interest in recent years as a key paradigm for understanding the complex interplay between heterogeneous psychopathological dimensions (Borsboom & Cramer, 2013), including trauma (Rossi et al., 2022) and psychosis (Misiak, Szewczuk-Bogusławska, Samochowiec, Moustafa, & Gawęda, 2023). More recently, Bayesian network analysis has emerged as an analytical method that provides a robust method for examining the associations between multiple disorder nodes from a causal relationship perspective, overcoming the long-standing limitation of non-directedness of conventional network models (Briganti, Scutari, & McNally, 2023). Given the conditional dependence between each couple of variables and according to the other variables in the network, it is possible to estimate the strength and direction of these associations. This approach allows for a nuanced understanding of the complex interdependencies and causal pathways among various disorders, facilitating more targeted and effective interventions.

Despite the consistent evidence showing that PTSD-related domains, such as cognitive schemas about the self, the world, and others, dissociation, and mood dysregulation are potential mediators between trauma and psychosis (Alameda et al., 2020; Sideli et al., 2020; Williams et al., 2018), none of the studies to date use a network approach. Elements of novelty in our study include a large representative sample of late adolescents, enriched with trauma exposure, and addressing in a network model all possible PTSD/cPTSD symptoms at once. Furthermore, our scope is to identify which specific post-traumatic stress symptoms are most likely responsible for driving sub-threshold psychotic experiences. Based on previous evidence, we postulate that cPTSD-related DSO will be most strongly associated with the emergence of PLEs. Finally, considering that elements such as gender (Comacchio et al., 2019) might influence this relationship, we seek to clarify the potential moderating effects of this variable when modeling the network structures.

## Materials and methods

### Participants and procedure

The present study is based on the Dual Trauma study cohort, a comprehensive epidemiological survey designed to explore the relationships between various traumatic exposures and behavioral and psychopathological outcomes in a non-clinical population. So far, dual trauma-related studies have focused on the association between traumatic experiences and substance use (Jannini et al., 2024; Rossi, R. et al., 2023c), psychotic-spectrum disorders (Rossi, R. et al., 2023b; Rossi, R. et al., 2023d), and non-suicidal self-injuries (submitted). In another study, the authors validated the Italian version of the International Trauma Questionnaire (Rossi et al., 2022). The study enlisted final-year students from ten high schools within the L’Aquila province, recruited between November 2019 and January 2020. Students who received special educational support were excluded to ensure that no cognitive impairments were present.

All participants were provided with detailed information about the study and gave their written consent. The research protocol was approved by the University of L’Aquila’s Institutional Review Board (local ethics committee) and adhered to the ethical guidelines stated in the Declaration of Helsinki.

### Measures

The primary instrument was the Italian version of the International Trauma Questionnaire (ITQ) (Rossi et al., 2022), an 18-item self-report questionnaire. From this, we focused on six PTSD-related symptoms (ptsd_1–6—see Supplementary Table 1) and six DSO-related symptoms (dso_1–6—see Supplementary Table 1), omitting the six items related to functional impact. These excluded items, three from the PTSD cluster (ptsd_7–9) and three from the DSO cluster (dso_7–9), were not designed to measure the severity of trauma symptoms but rather the effect of trauma on daily functioning. Thus, we decided to rule them out from the statistical analyses. Participants responded using a 1 to 4 Likert scale. Detailed diagnostic algorithms are available on the official website of the instrument (https://www.traumameasuresglobal.com/itq).

The Italian version of the Prodromal Questionnaire-16 (iPQ-16) was used to evaluate the presence of PLEs (Azzali et al., 2018). The iPQ-16 is a self-report tool consisting of 16 items that examine the presence or absence of 16 PLEs, such as perceptual aberrations/hallucinations (e.g. “*I sometimes smell or taste things that other people can’t smell or taste*,” or “*When I look at a person, or look at myself in a mirror, I have seen the face change right before my eyes*”), unusual thought content/delusions (e.g. “*My thoughts are sometimes so strong that I can almost hear them*,” or “*I sometimes see special meanings in advertisements, shop windows, or in the way things are arranged around me*”), and negative symptoms (i.e. “*I feel uninterested in the things I used to enjoy*”). The instrument scores the number of actual PLEs endorsed, ranging from 0 to 16, and includes a distress score on a 4-point Likert scale, ranging from 0 to 48. Originally designed as a screening tool for ultra-high-risk individuals in help-seeking populations, the iPQ-16 has been widely used in the general population to measure PLEs (Gawęda, Göritz, & Moritz, 2019; Gawęda et al., 2020). Scores were used as continuous or categorical variables (using ≥6 as the cutoff value) (Savill, D’Ambrosio, Cannon, & Loewy, 2018).

### Data analysis

We first provided univariate descriptive statistics of whole sample characteristics, with the prevalence of probable PTSD/ cPTSD, and PLEs (Table 1), broken down by gender (Supplementary Table 2).

**Table 1.** Sample characteristics

Variable	Median (IQR)/numerosity (%)
	*N* = 981
*Age*	18.50 (18.33–18.84)
*Gender*	
Female	481 (49%)
Male	492 (50%)
Prefer not to say	9 (0.9%)
*Education*	
Lyceum	506 (52%)
Technical	462 (47%)
Vocational	13 (1.3%)
*Trauma diagnosis*	
probable PTSD	90 (9.2%)
probable cPTSD	41 (4.2%)
*PLEs diagnosis*	
PLEs	416 (42%)
no PLEs	565 (58%)

Legend - IQR = interquartile range; PTSD = post-traumatic stress disorder; cPTSD = complex PTSD; PLEs = psychotic-like experiences

We then analyzed the network of PTSD/cPTSD symptoms and PLEs (detailed descriptions of each node are available in Supplementary Table 1). To do this, we used a Gaussian graphical model structured around partial correlations. This model estimates the connection between each pair of symptoms (nodes) while controlling for the severity of all other symptoms in the network. Stronger connections are depicted with thicker edges, colored green for positive and dashed red for negative associations. The networks were estimated using the regularized graphical LASSO procedure, which removes unnecessary connections based on the extended Bayesian information criterion (Epskamp, Borsboom, & Fried, 2018).

To identify bridging connections between PTSD symptoms and PLEs, we calculated the bridge strength and the bridge’s expected influence within this network. The bridge strength of a node is the sum of the absolute weights of its connections spanning different clusters, indicating how many connections a node typically shares across multiple clusters (PTSD, DSO, and PLEs). Similarly, the bridge’s expected influence measures the sum of the values (positive or negative) of all edges between a given node and all nodes outside its cluster, reflecting the direction of influence a node exerts on other clusters.

According to the network theory of mental disorders, bridge strength quantifies a node’s overall role in linking disparate clusters within the network. In contrast, bridge expected influence provides insights into the directional impact of a node beyond its immediate community, highlighting its significance in connecting different clusters within the network.

Once the overall structure was estimated, we computed different network analyses according to gender. We then performed the Network Comparison Test (NCT) to report any differences in network invariance and global strength between networks. This test allows to compare the structural properties and overall connectivity of different networks, providing insights into whether and how these networks differ significantly (van Borkulo et al., 2023).

Finally, we aimed to determine the potential causal relationship between PTSD/cPTSD and PLEs by reanalyzing the data using a directed acyclic graph (DAG). Based on a Bayesian approach, this method is a recent addition to network analyses. Unlike undirected networks, Bayesian DAGs are designed to identify and represent the most likely direction of causal relationships between symptoms by analyzing the conditional dependencies between variables.

For this analysis, we followed procedures outlined in a recent study (Briganti et al., 2023). The process involves “learning” the network structure using a hill-climbing algorithm, which iteratively computes the network’s structure (n = 1,000 iterations) and evaluates each edge’s goodness-of-fit using the Bayesian Information Criterion (BIC). The final averaged network is selected by applying a validated threshold-based method to optimize sensitivity and specificity, retaining only those edges and causal directions that consistently appear in a substantial proportion of iterations defined by the threshold.

To facilitate interpretation, edges that appeared in more than 85% of the networks (a parameter called strength) and whose direction appeared in 50% or more of the iterations (called minimum direction) were plotted as directed (with arrows), while the remaining edges were plotted as undirected. The averaged network was visualized by weighing the shade of each edge by its arc strength to indicate the relative importance of each edge in the network. Arc strength reflects the magnitude of the corresponding BIC value for each edge, so a large negative value for an edge suggests that removing it would significantly worsen the network’s fitness.

Finally, the same set of analyses was reproduced in the male and female subpopulations. This was done to graphically inspect for differences in edge causal relationships across genders.

All analyses were performed using R (R Core Team, 2021). Data wrangling was performed using *tidyverse*, version 2.0 (Wickham et al., 2019). The networks were estimated and visualized using the *qgraph* package, version 1.9.8 (Epskamp, Cramer, Waldorp, Schmittmann, & Borsboom, 2012). Bridge centrality metrics were computed with the *networktools* package, version 1.5.1 (Jones & Jones, 2018). The accuracy and stability of the networks (defined with stability indices ≥0.25) were computed with the *bootnet* package, version 1.5.6 (Epskamp et al., 2018). The DAG was estimated using the package *bnlearn*, version 4.9.4 (Scutari, 2009).

## Results

One thousand and ten individuals participated in the study. Twentynine participants reported missing data and therefore were excluded from further analyses. Table 1 reports the characteristics of the 981 adolescents who took part in the study. Supplementary Table 2 reports the study sample’s characteristics as broken down by gender.

### Network of post-traumatic stress symptoms, disturbances of self-organization, and psychotic-like experiences

**Figure 2.**
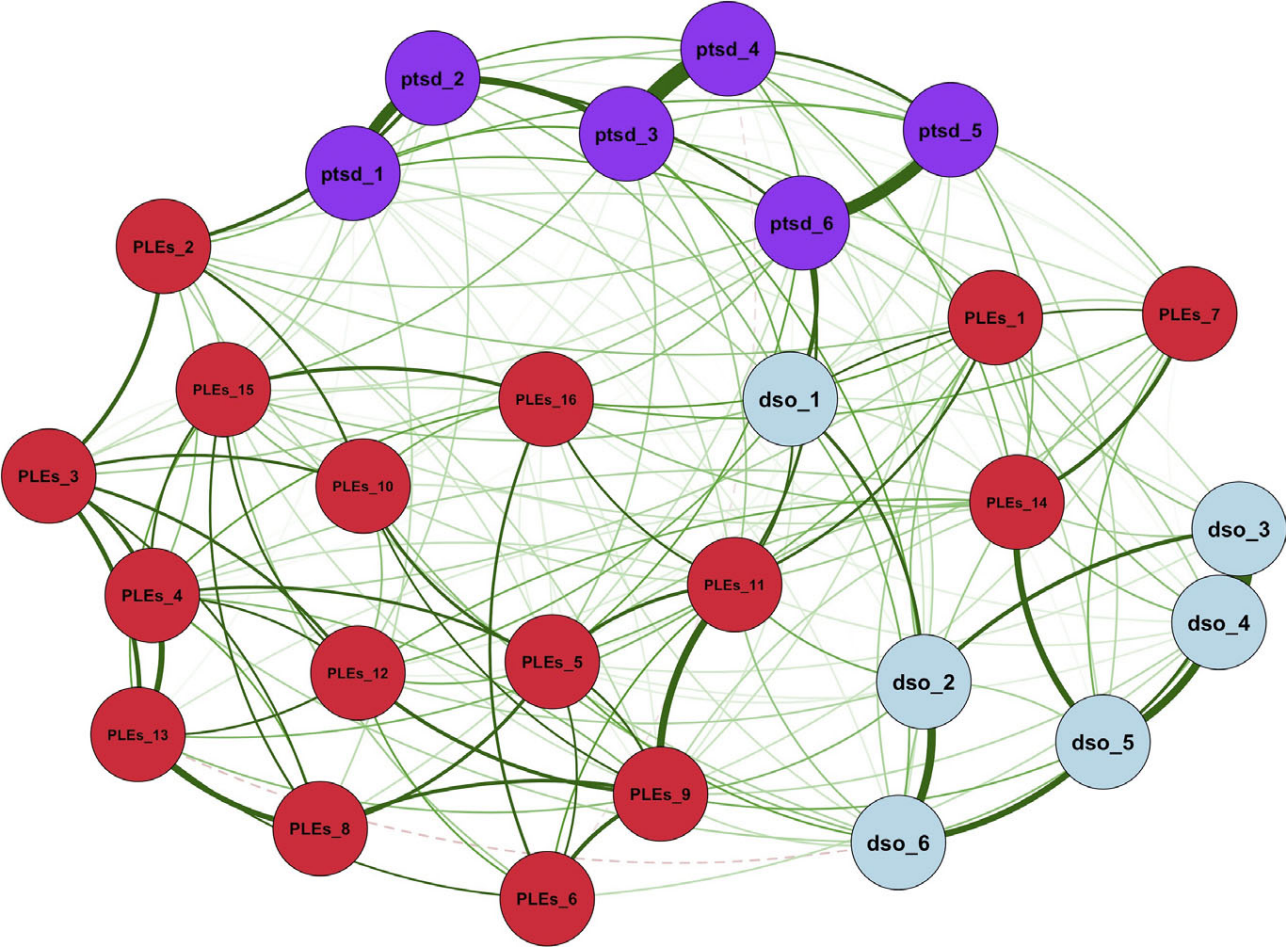
Network Structure of trauma-related symptoms and psychotic-like experiences. Legend - PTSD (purple): Post-traumatic stress disorder; DSO (light blue): Disturbances of self-organization; PLEs (red): psychotic-like experiences. Continuous green edges indicate a positive correlation between nodes, whereas dashed red edges indicate a negative correlation; thicker lines indicate a stronger correlation. Reference for nodes: ptsd_1 - ptsd_2: Re-experiencing symptoms; ptsd_3 - ptsd_4: avoidance symptoms; ptsd_5 - ptsd_6: hyperarousal symptoms; dso_1 - dso_2: affective dysregulation; dso_3 - dso_4: negative self-concept; dso_5 - dso_6: problems in interpersonal relationships; PLEs_9–11, PLEs_14, PLEs_15: unusual thought content; PLEs_2–6, PLEs_8, PLEs_12, PLEs_13, PLEs_16: perceptual aberrations; PLEs_7: social anxiety; PLEs_1: negative symptoms. The LASSO correction has been applied to build this graph.

**Figure 3.**
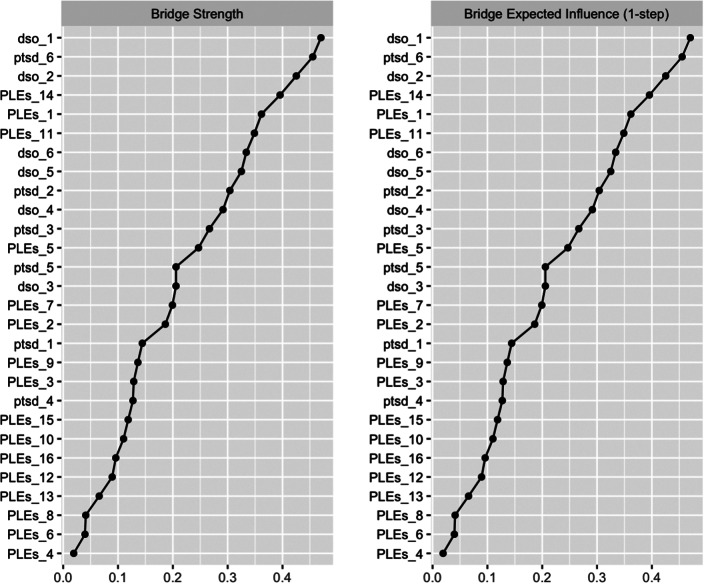
Bridge centrality indices for the proposed network structure.Legend - PTSD: Post-traumatic stress disorder; DSO: disturbances of self-organization; PLEs: psychotic-like experiences. Reference for nodes: ptsd_1 - ptsd_2: Re-experiencing symptoms; ptsd_3 - ptsd_4: avoidance symptoms; ptsd_5 - ptsd_6: hyperarousal symptoms; dso_1 - dso_2: affective dysregulation; dso_3 - dso_4: negative self-concept; dso_5 - dso_6: problems in interpersonal relationships; PLEs_9–11, PLEs_14, PLEs_15: unusual thought content; PLEs_2–6, PLEs_8, PLEs_12, PLEs_13, PLEs_16: perceptual aberrations; PLEs_7: social anxiety; PLEs_1: negative symptoms. The LASSO correction has been applied to build this graph.

The network of PTSD/cPTSD symptoms and PLEs contained 398 edges (Figure 2). The network presented good stability indices (strength and expected influence = 0.67 – Supplementary Figure 1). Stability indices help assess how stable and unchanged the overall network configuration remains when individual connections or nodes are altered or removed. Higher stability coefficients denote a more stable network (the cutoff is generally accepted as ≥0.25 (Epskamp et al., 2018)). The node that reported the highest bridge centrality indices was DSO item 1 (*When I am upset, it takes me a long time to calm down –* bridge strength = 0.470; bridge expected influence = 0.470 – Figure 3), pertaining affect dysregulation. Bridge centrality estimates indicate the capacity of a single node in a specific cluster to establish connections with other nodes in other clusters. The strongest bridge connections that the latter node reported were with PLEs item 11 (*Sometimes I have felt that I’m not in control of my own ideas or thoughts* – edge weight = 0.081) and with PLEs item 7 (*I get extremely anxious when meeting people for the first time* – edge weight = 0.079).

### Role of gender as moderator

We then compared the network structures of PTSD/cPTSD between subgroups of participants divided into two levels according to gender.

When considering gender as a possible moderator (492 vs 480 individuals, Supplementary Table 2), analyses revealed two stable networks (Supplementary Figures 2–5) as well as a significant difference in the overall connection strength (Supplementary Figures 6 and 7). The global strength for the males’ network was 5.00, while for the females’ network was 11.91 (global strength invariance test S = 6.904, p < 0.001). Both networks revealed good stability of the centrality indices (males’ strength and expected influence = 0.595; females’ strength and expected influence = 0.516, Supplementary Figures 1 and 3). The difference in the overall connection strength between networks can also be seen in Supplementary Figures 2 and 4, which show increased connectivity between nodes in the females compared to males’ network. The two networks also presented differences in bridge centrality indices. Among males, the node with the highest bridge strength and expected influence was DSO item 5 (*I feel distant or cut off from people* – bridge strength and expected influence = 0.345, Supplementary Figure 6), while females showed DSO item 1 as the most central (*When I am upset, it takes me a long time to calm down* —bridge strength and expected influence = 0.470, Supplementary Figure 7).

### Bayesian network analysis – directed acyclic graphs (DAGs)

**Figure 4.**
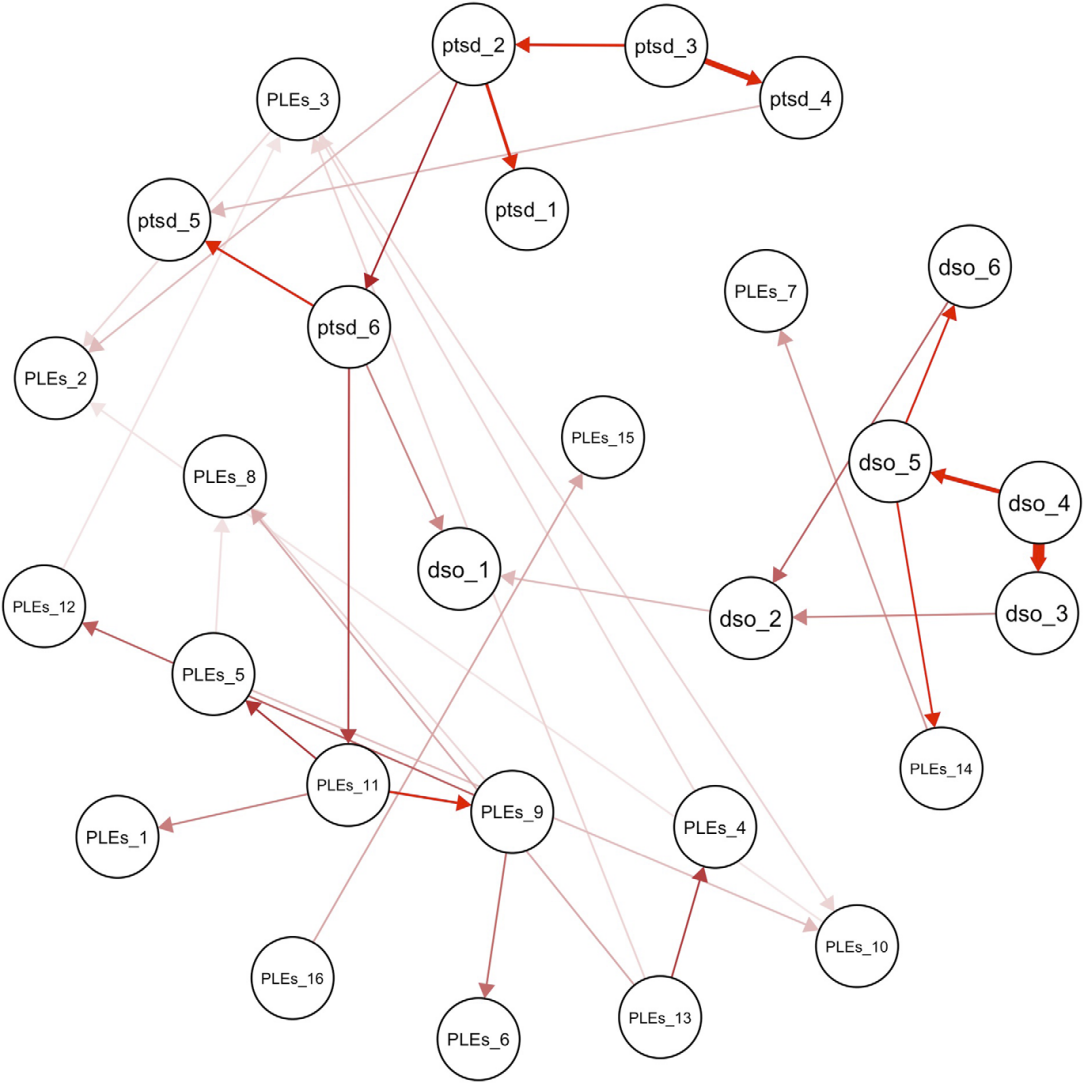
Directed acyclic graph (DAG) of trauma-related symptoms and psychotic-like experiences.Legend - PTSD: Post-traumatic stress disorder; DSO: disturbances of self-organization; PLEs: psychotic-like experiences. Red arrows indicate a causal relationship. Arrow shade refers to arc strength, with more intense colors indicating higher relative importance of each edge in the network. Reference for nodes: ptsd_1 - ptsd_2: Re-experiencing symptoms; ptsd_3 - ptsd_4: avoidance symptoms; ptsd_5 - ptsd_6: hyperarousal symptoms; dso_1 - dso_2: affective dysregulation; dso_3 - dso_4: negative self-concept; dso_5 - dso_6: problems in interpersonal relationships; PLEs_9–11, PLEs_14, PLEs_15: unusual thought content; PLEs_2–6, PLEs_8, PLEs_12, PLEs_13, PLEs_16: perceptual aberrations; PLEs_7: social anxiety; PLEs_1: negative symptoms. The LASSO correction has been applied to build this graph.

The procedure by Briganti et al. identified 0.85 as the optimal significance threshold for strength (Briganti et al., 2023). Thus, edges appearing in 85% or more of the bootstrapped networks were retained in the averaged DAG of PTSD/cPTSD and PLEs. In the DAG (Figure 4), only those edges with an unequivocal minimum direction (50% or more iterations displaying the same direction) were plotted as directed.

The Bayesian network analysis on the overall sample revealed a distinct pathway connecting cPTSD symptoms with PLEs. Results displayed the largest strength of association, the highest probability of causal relationship between nodes (minimum direction), and the highest importance of each edge in the network (arc strength). This pathway connects DSO item 4 (*I feel worthless*) with DSO item 5 (*I feel distant or cut off from people* – strength = 0.999, minimum direction = 0.739, arc strength = −231.71), which was then connected with PLEs item 14 (*I often feel that others have it in for me*, strength = 0.999, minimum direction = 0.851, arc strength = −102.36), which finally is connected with PLEs item 7 (*I get extremely anxious when meeting people for the first time*, strength = 0.951, minimum direction = 0.910, arc strength = −38.71).

Males and females presented DAGs similar to the overall sample except for a few edges. Among males, findings show the same pathway as the overall population, that is the one going from DSO item 4 and item 5 to PLEs item 14 and item 7. The female DAG features instead DSO item 3 (*I feel like a failure*) as the causative node of a similar pathway (i.e. dso_3 ➔ dso_4 ➔ dso_5 ➔ PLEs_14). In particular, DSO item 3 shows a causal relationship with DSO item 4 (strength = 1.0, minimum direction = 0.508, arc strength = −264.97). See Supplementary Figure 8 and Supplementary Tables 5–8.

## Discussion

### Main findings

This study investigated for the first time the relationship between post-traumatic stress symptoms and PLEs among 1010 adolescents adopting a Gaussian and Bayesian network approach. Non-clinical representative samples are precious in psychosis research as psychotic phenomena are thought to exist on a continuum with normal experiences. Additionally, non-clinical representative samples eliminate the confounding effects of psychosis treatment or diagnosis, providing clearer insights into the phenomena under investigation.

The analysis revealed several key findings regarding the structure and stability of these symptom networks and the role of gender as a moderator. As expected, disturbances in self-organization, that is affect dysregulation, negative self-concept, and problems in interpersonal relationships, were crucial both for sustaining the comorbidity and for transitioning to psychotic-like experiences.

In particular, the finding of DSO “affect dysregulation” as the node with the highest bridge centrality index in the undirected network underscores the interconnectedness of this complex post-traumatic stress symptoms domain with PLEs. This is consistent with several studies that emphasize the role of affect dysregulation in linking trauma-related symptoms to psychotic experiences (Lincoln, Marin, & Jaya, 2017; Marwaha, Broome, Bebbington, Kuipers, & Freeman, 2014; van Nierop et al., 2014). For instance, a recent longitudinal study (Bornheimer et al., 2022) as well as a metanalytical study (Panayi et al., 2022) showed either the direct relationship between affective dysregulation and PLEs or its mediational effect between trauma and psychosis.

However, the undirected network analysis results should not be interpreted as establishing causation. Instead, the centrality of emotional instability should be understood as evidence that this domain significantly correlates with unusual subthreshold experiences and contributes to their persistence over time, acting as a maintaining factor (Garety, Bebbington, Fowler, Freeman, & Kuipers, 2007). This means that having symptoms like mood swings, overly intense emotions, or lack of emotional awareness probably contributes to the persistence of PLEs over time or even amplifies their frequency and severity although without necessarily being the primary cause of them. In this regard, emotional dysregulation has been widely described as a secondary mediator or facilitating factor, rather than a direct driver, for PLEs or other mental disorders (Akram et al., 2020; Fernández, Vallina-Fernández, Alonso-Bada, Rus-Calafell, & Paino, 2024; Grady, Twomey, Cullen, & Gaynor, 2024; Peters, Yates, DeVylder, Lodhi, & Kelleher, 2022). Failure to regulate emotions may be related to the loosening of the sense of agency/ownership of thoughts (PLEs item 11) connected with distressing trauma-related memories and heightened anxiety and difficulties in social interactions (PLEs item 7) (Mertin & O’Brien, 2013). This indicates that affect dysregulation plays a pivotal role in the interplay between trauma-related symptoms and PLEs and poses important implications for further therapeutic interventions. Overall, these findings on affective dysregulation contribute to the “Affective Pathway to Psychosis” hypothesis, which postulates that mood-related attributes such as anxiety and mood dysregulation and subthreshold depression may put reality testing under pressure in traumatized vulnerable individuals, contributing to psychosis risk (Bebbington, 2015; Myin-Germeys & van Os, 2007).

Moderation analyses revealed that gender exhibited distinct associations between post-traumatic stress symptoms and PLEs. This finding indicates that the impact of post-traumatic stress symptoms on PLEs, and *vice versa*, varies significantly between males and females, suggesting that gender-specific factors play a crucial role in this relationship. Firstly, results showed a more densely connected network structure among females compared to males. This is further in line with the Affective Pathway to Psychosis hypothesis (Isvoranu et al., 2017; Myin-Germeys & van Os, 2007) as well as the most recent literature, which shows, besides a higher prevalence of certain types of trauma, a greater association between post-traumatic stress symptoms and PLEs among females in community samples of adolescents (Comacchio et al., 2019; Fisher et al., 2009). Possible explanations might be found in genetic variations and/or in hormonal factors, as suggested by animal studies (Barr et al., 2004). Women also tend to perceive traumatic events as more threatening and stressful compared to men, which may increase their susceptibility to dysregulation of the hypothalamic–pituitary–adrenal axis following a major trauma (De Bellis et al., 1994). This heightened sensitivity could contribute to a greater risk of developing psychosis later in life (Comacchio et al., 2019).

Further network differences were also reported in bridge centrality symptoms, with problems in interpersonal relationships being more central in males, and affect dysregulation being more central in females. These results are consistent with the established literature, which shows males to be more prone to externalizing behaviors and social interaction issues in response to traumatic events, and females to be more susceptible to internalizing symptoms, including emotional instability (Søegaard, Kan, Koirala, Hauff, & Thapa, 2021).

The Bayesian network analysis reported a specific pathway connecting cPTSD symptoms to PLEs, with negative self-concept leading to disturbances in interpersonal relationships, leading to paranoic thoughts, and finally to social anxiety. The primary scope of a DAG is to show the map of conditional independence between variables, with arrow-connected nodes expressing a significant causal relationship. Our pathway seems distinctive not only because it appears plausible but also because of the largest values of its fit parameters, like minimum direction and arc strength, expressing the relative importance of a specific association within the DAG. The link between feelings of worthlessness and social disconnection is well-documented in the literature. A study by Cloitre et al. found that negative self-concept significantly impacts social relationships (Cloitre et al., 2011). The further transition from social disconnection (or loneliness) to paranoia aligns with findings from the most recent evidence, which demonstrated that social isolation could lead to increased suspicion and paranoic ideation (Lamster, Nittel, Rief, Mehl, & Lincoln, 2017; Monsonet, Amedy, Kwapil, & Barrantes-Vidal, 2023). This is particularly relevant in individuals with cPTSD, who may already have heightened sensitivity to social threats due to past traumas (Di Lorenzo et al., 2020; Simon, Roberts, Lewis, van Gelderen, & Bisson, 2019). Such heightened sensitivity can contribute to developing negative schemata about others, where individuals perceive others as threatening, untrustworthy, or malevolent.

Both paranoia and social anxiety involve the “fear of the other” and often co-occur, thus explaining the arrowed edge connecting these two nodes (Schutters et al., 2012). The individual might delusionally believe he/she is the focus of negative attention, potentially due to overestimating the significance of his/her own actions and engaging in self-referential and confirmatory biases. In this regard, Clark and Wells’ cognitive model posits that social phobia is grounded in near-delusional thoughts about oneself and the social environment (Clark & Wells, 1995). It, therefore, seems easy to understand how perceiving others as threatful might lead to shifting the attention towards close monitoring and observing oneself, thus diminishing one’s capacity to process external information and finally leading to social anxiety. Consistently, the longitudinal population-based NEMESIS study showed a significant association between paranoid thoughts and the subsequent onset of social phobia (Rietdijk, Van Os, de Graaf, Delespaul, & van der Gaag, 2009). The “poor me-bad me” distinction also offers insights into the link between worthlessness, paranoia, and social anxiety. In “poor me” paranoia, externalizing blame aligns with self-referential biases seen in social anxiety, while “bad me” paranoia, rooted in internalized guilt, exacerbates feelings of worthlessness. (Trower & Chadwick, 1995). The instability of these subtypes, as noted by Melo et al., highlights how shifts between external blame and self-criticism may reinforce both paranoia and social anxiety (Melo, Taylor, & Bentall, 2006).

Although it has been largely debated how trauma-related disorders and cluster B personality disorders are distinct nosological entities (Leichsenring et al., 2024), the connection between DSO and PLEs shares significant theoretical overlap with Kernberg’s concept of borderline personality organization. According to Kernberg, individuals with borderline traits are characterized by deficits in emotional regulation, unstable self-concept, and difficulties in interpersonal relationships, which parallel the DSO components of cPTSD (Leichsenring et al., 2024). These features are often accompanied by transient psychotic-like experiences, particularly under conditions of stress or trauma (Baryshnikov, Aaltonen, et al., 2018; Sengutta, Gawęda, Moritz, & Karow, 2019).

When considering gender as a moderating variable also in the Bayesian network analysis, results showed a slightly different DAG in females compared to males. Our findings reported the node indicating feelings of failure as the apex of the relationship between complex post-traumatic stress symptoms and PLEs in the female population. This difference highlights the central role of self-perception in females, where feelings of failure significantly contribute to feelings of worthlessness, which in turn lead to paranoia and social anxiety. Once again, this is consistent with the literature on gender differences in response to trauma, which suggests that females are more likely to internalize distress and experience heightened self-criticism and feelings of failure (Søegaard et al., 2021).

### Implications and future Research

The study presents several significant implications for understanding PLEs in adolescents, particularly when associated with post-traumatic stress symptoms /complex post-traumatic stress symptoms.

The centrality of emotional instability suggests that therapeutic interventions focusing on affect regulation may be particularly effective in mitigating PLEs in adolescents with complex post-traumatic stress symptoms. Besides pharmacological interventions (Jannini et al., 2022), psychological techniques such as Emotion Regulation Therapy (ERT) or Eye Movement Desensitization and Reprocessing (EMDR) that address affect dysregulation might be beneficial when tailored according to the specific type of trauma and specific clinical picture, taking into account the difficulty and the potential harms of addressing complex trauma with such techniques (Karatzias et al., 2019; Varese et al., 2024). In particular, a recent systematic review and meta-analysis highlighted the significant role of exposure therapies (i.e. EMDR) compared to non-exposure therapies in reducing psychotic symptoms following traumatic experiences (Reid, Cole, Malik, Bell, & Bloomfield, 2024).

Given the gender differences observed, treatment approaches should be tailored to address the specific needs of males and females. For females, interventions might focus more on managing internalizing symptoms and emotional instability, while for males, improving interpersonal relationships and addressing externalizing behaviors could be more effective (Søegaard et al., 2021).

The identification of a specific pathway from negative self-concept to social anxiety through problems in interpersonal relationships and paranoia underscores the importance of early intervention. Preventative measures and early therapeutic interventions targeting self-esteem, social skills, and paranoia could help reduce the progression from traumatic experiences to more severe PLEs.

The interconnectedness of complex post-traumatic stress symptoms and PLEs suggests that a holistic treatment approach addressing multiple domains of functioning—emotional, social, and cognitive—might be more effective than approaches focusing on isolated symptoms.

The study’s findings underscore the need for longitudinal research further to elucidate the causal relationships between complex post-traumatic stress symptoms and PLEs. Such studies could help identify critical periods for intervention and better understand the developmental trajectories of these symptoms.

### Limitations

This study has limitations, firstly due to its reliance on self-report data, which can introduce recall bias, especially when participants recount past traumatic experiences. Furthermore, when analyzing data from adolescent populations, it is important to consider the possibility of intentional, careless, or confused inaccurate responses, which can complicate the interpretation of results (Fan et al., 2006). This might be particularly true for the item 7 and 11 of the iPQ-16 questionnaire. The first one may indeed capture a transient discomfort in social settings rather than the persistent fear or avoidance characteristic of clinical social anxiety. Similarly, the iPQ-16 item 11, besides expressing a loss of self-agency, it may also capture other cognitive or emotional states, such as intrusive thoughts or general cognitive disorganization, which could be less specific to psychosis (Ossola, Fortunati, Marchesi, Rossi, & Maggini, 2020). Social desirability bias may also be particularly prevalent among young adults, leading to potential inaccuracies in self-reported data (Latkin, Edwards, Davey-Rothwell, & Tobin, 2017).

Additionally, even though the DAGs indicate the most likely causal direction of symptom interactions, they are based on cross-sectional data, necessitating caution when interpreting these findings, which should be intended, at best, as exploratory. The assumption of acyclicity inherent in DAG modeling may be particularly challenging to justify in the context of psychological processes, where bidirectional or cyclical interactions may often subsist. Thus, while DAGs provide valuable insights into potential causal structures, their reliance on assumptions that may not fully capture the complexity of these interactions limits their interpretive power. Future research employing longitudinal or experimental designs is critical to validate the causal relationships suggested by these models. Such studies could better account for temporal dynamics and allow for a more robust investigation of causality in symptom interactions.

Finally, while our findings highlight a pathway from cPTSD to PLEs, it is important to note that specificity cannot be fully established. Other unmeasured variables, such as depressive symptoms, obsessive-compulsive traits, substance use, or other symptom dimensions, could also contribute to or explain this relationship. As our study was limited by the scope of the administered questionnaires, alternative pathways connecting trauma to psychotic-like experiences cannot be ruled out.

## Conclusions

Our results are in line with the continuum model of trauma-psychotic experiences. They indeed indicate a dramatic contribution of complex post-traumatic stress symptoms domains of affect dysregulation, negative self-concept, and problems in interpersonal relationships in explaining the comorbidity with PLEs among adolescents. Furthermore, these relationships are shaped by gender, with females relying on internalizing and males on externalizing symptoms as proxies of unusual subthreshold experiences. Our study warrants the potential utility of psychotherapeutic interventions focusing on these symptoms to prevent the onset of quasi-psychotic symptoms in those with trauma and, therefore, further worsen clinical pictures among adolescents.

## Supporting information

Jannini et al. supplementary material Jannini et al. supplementary material

## Supplementary material

To view supplementary material for this article, please visit http://doi.org/10.1017/S0033291725000169.
