# Supplementary material for: Mapping connections between complex post-traumatic stress disorder and psychotic-like experiences among adolescents: a Gaussian and Bayesian network study
Source: Psychol Med. 2025 Feb 25;55:e61. doi: 10.1017/S0033291725000169 (PMC12080637; doi:10.1017/S0033291725000169)
Supplement: Jannini et al. supplementary material [file S0033291725000169sup001.docx]

**Supplementary Material**

***Supplementary Figure 1 - Stability of the Centrality Indices of the overall network model***

**
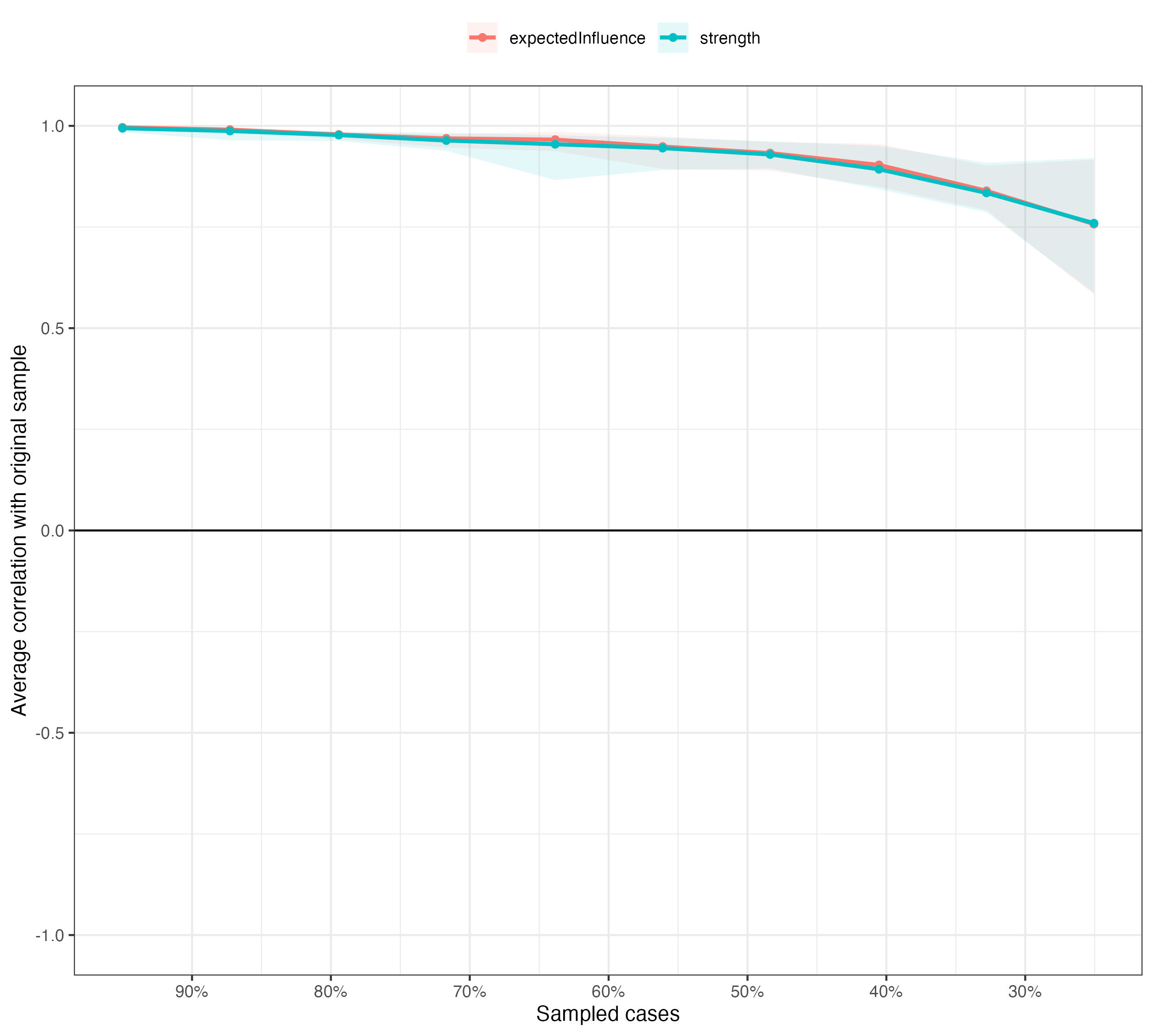
**

***Supplementary Figure 2 - Male Network***

***
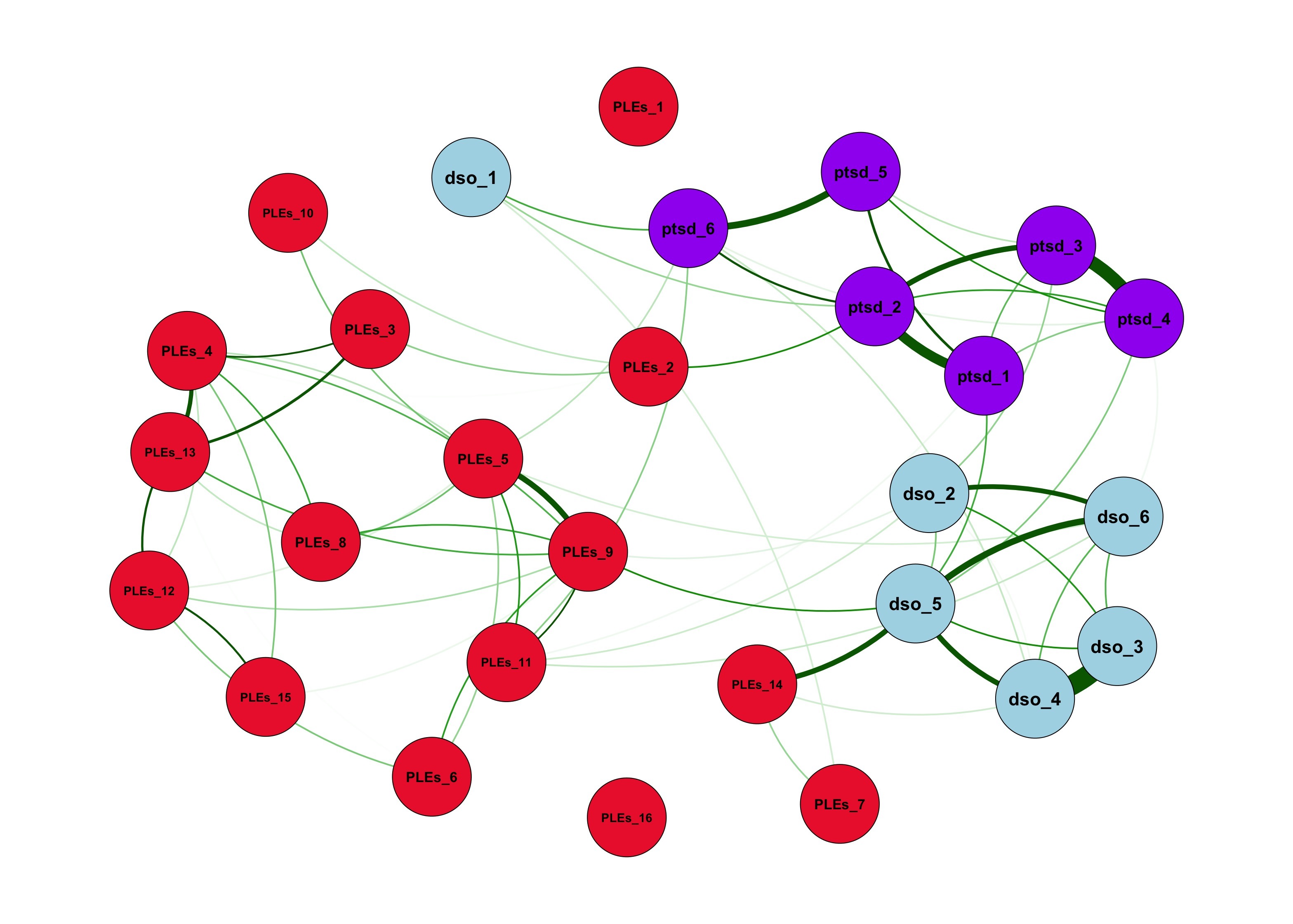
***

***Supplementary Figure 3 - Stability of the Centrality Indices of the Males' Network***

***
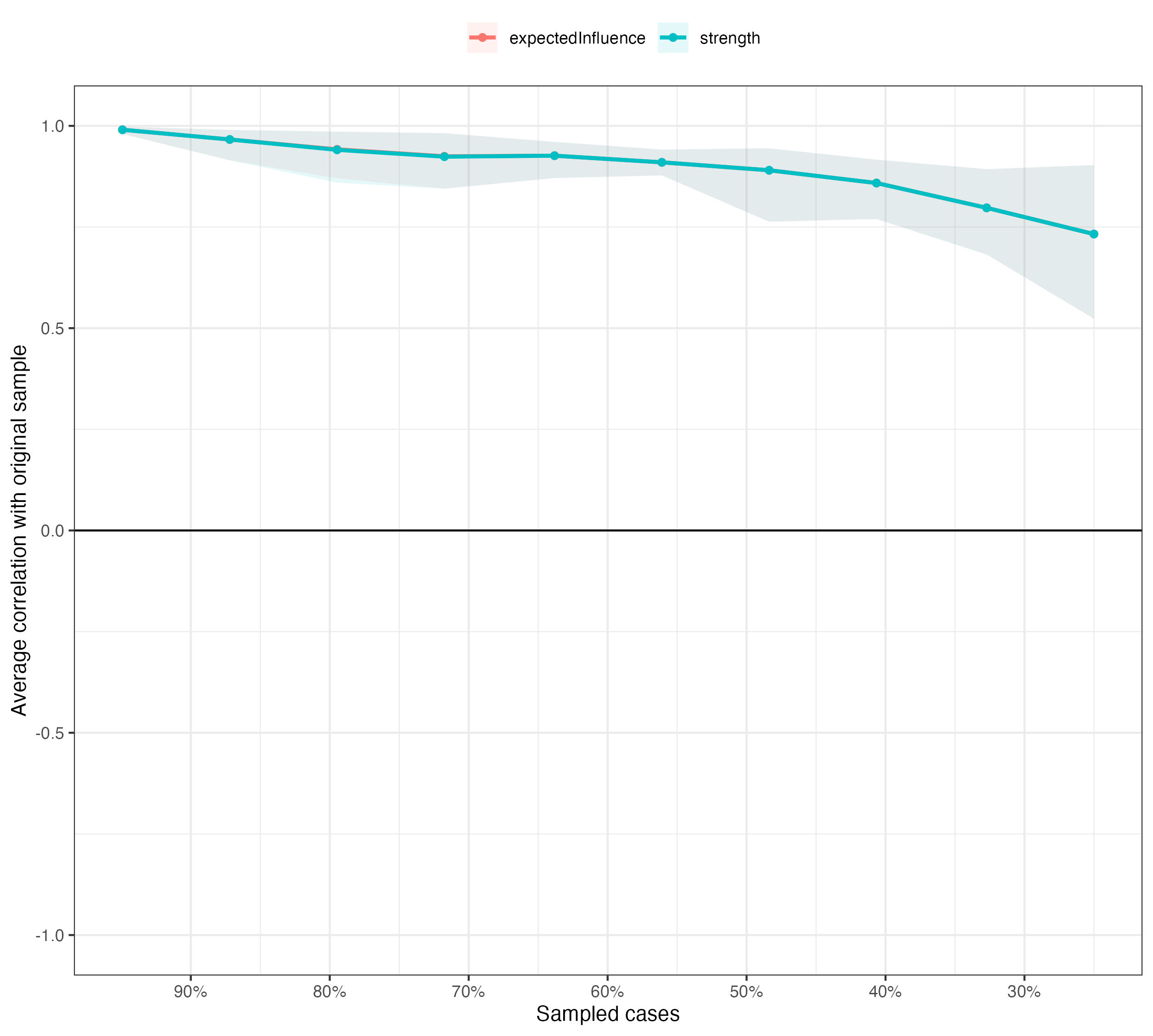
***

***Supplementary Figure 4 – Females’ Network***

***
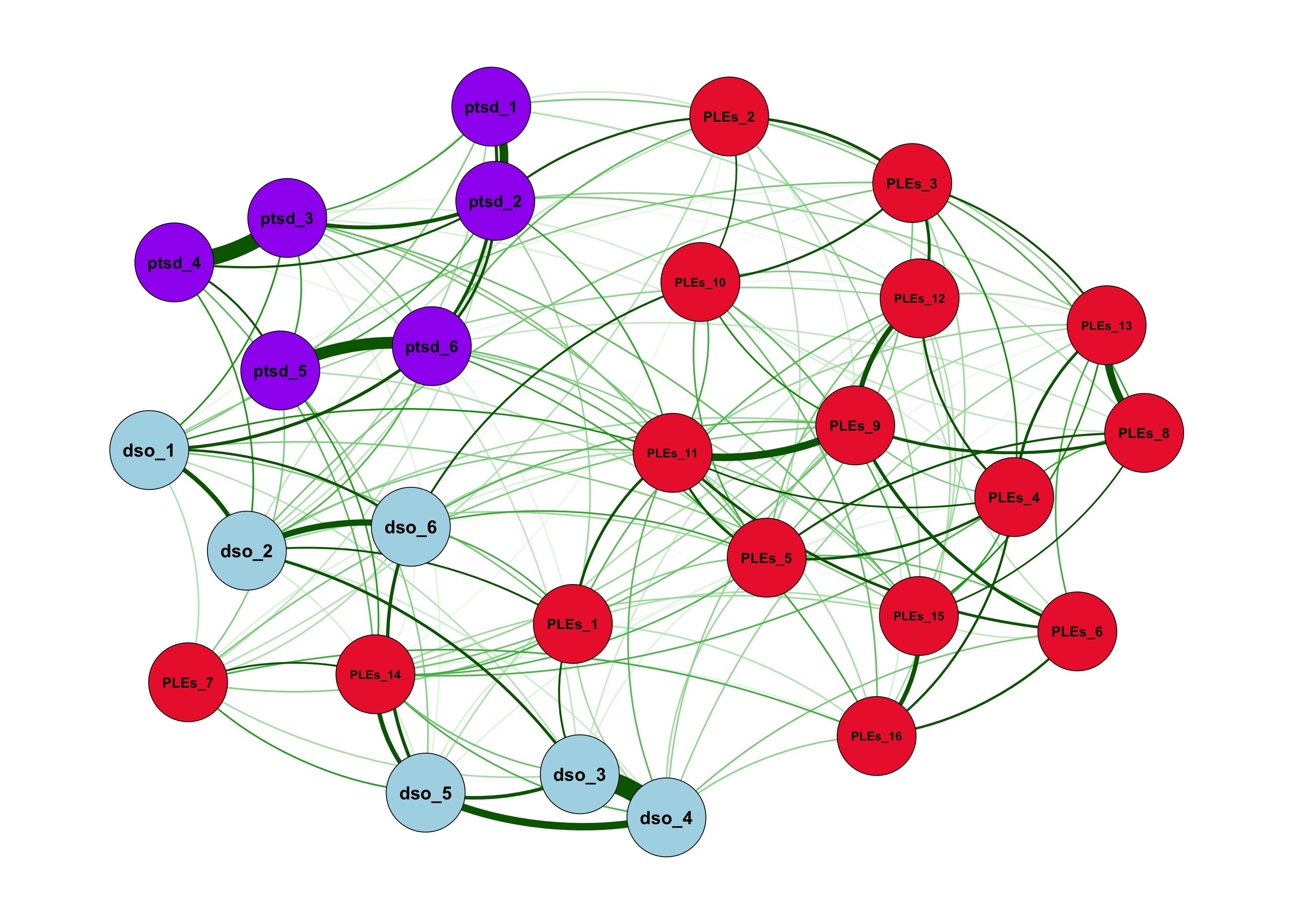
***

***Supplementary Figure 5 - Stability of the Centrality Indices of the Females' Network***

***
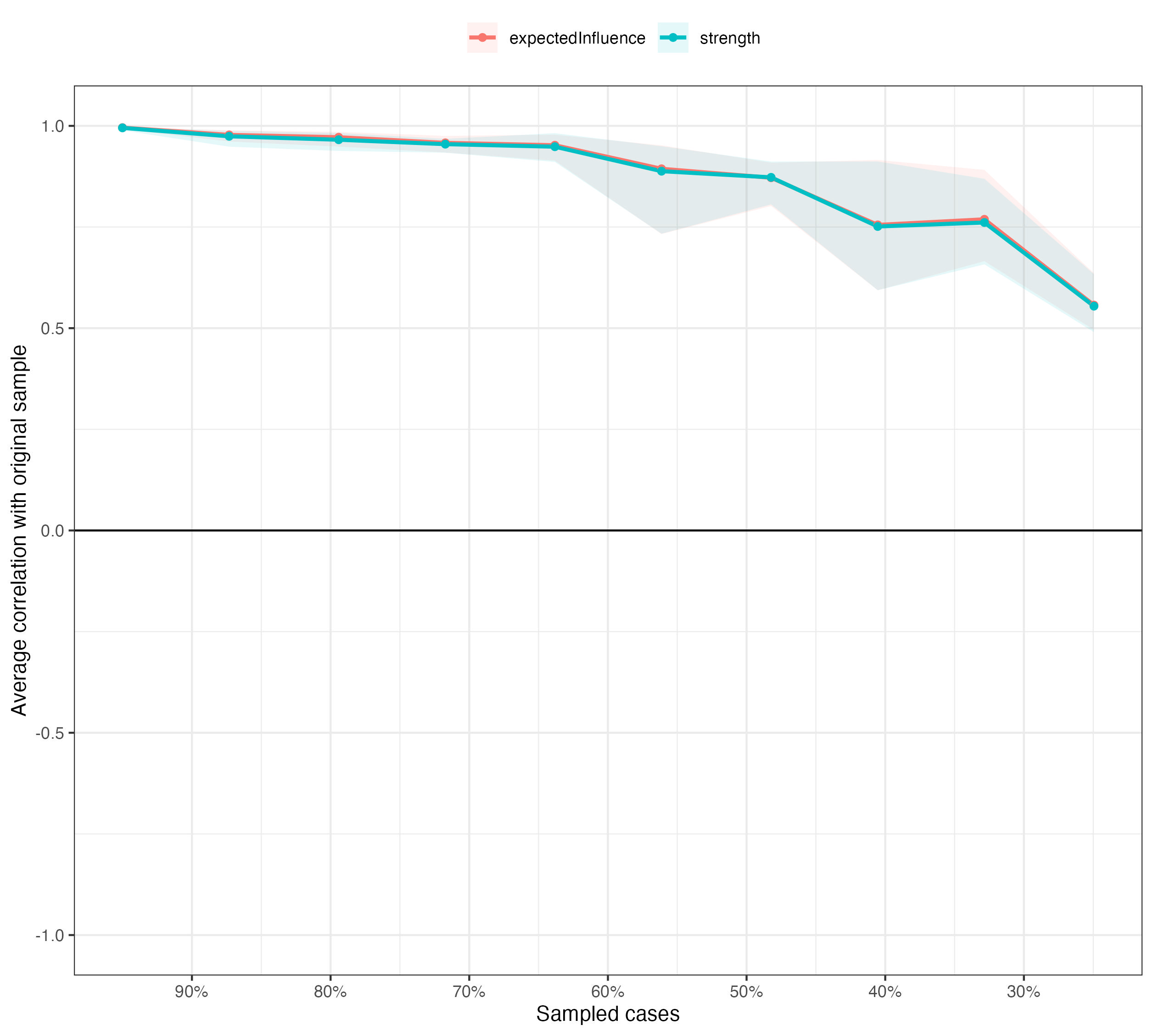
***

***Supplementary Figure 6 - Bridge Centrality Indices for Males' Network***

***
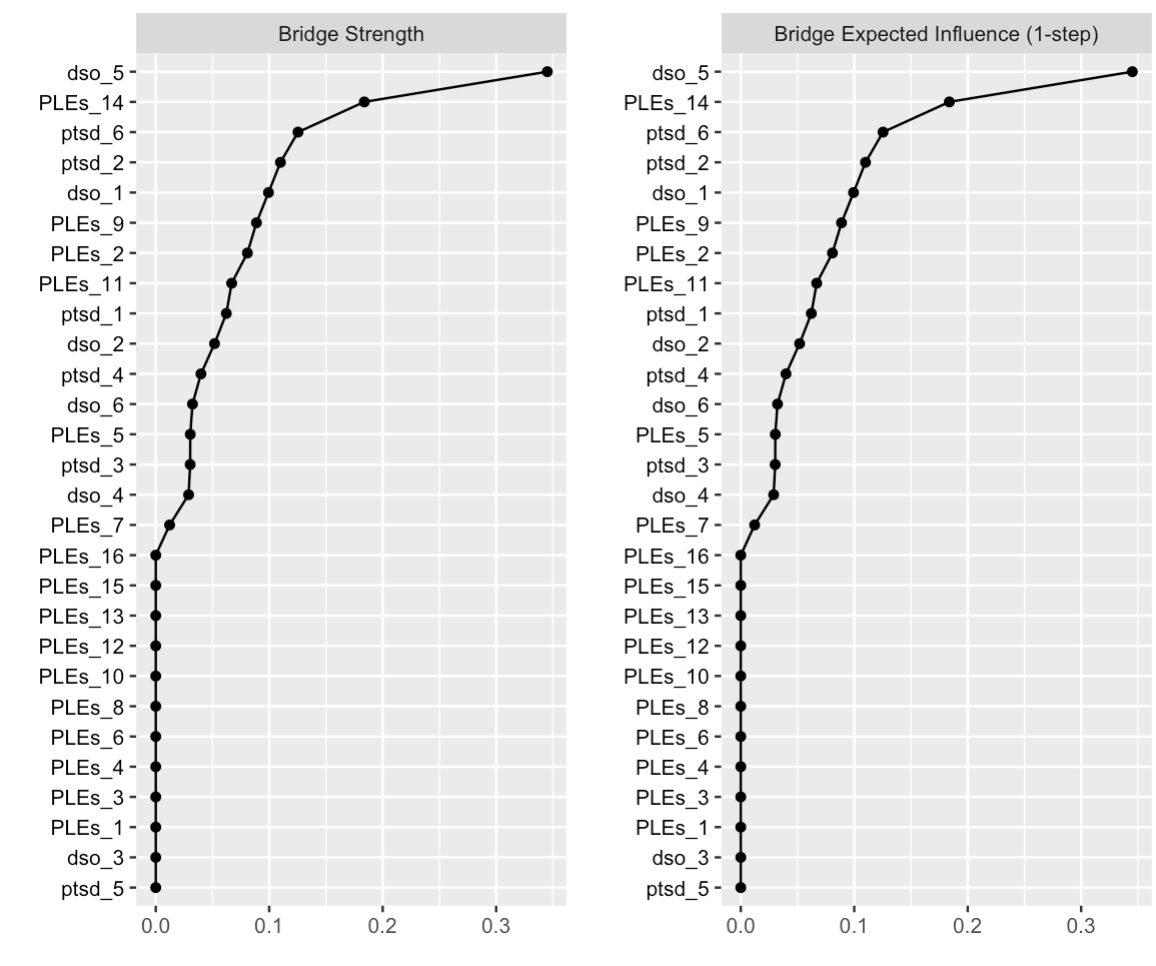
***

***Supplementary Figure 7 - Bridge Centrality Indices for Females' Network***

***
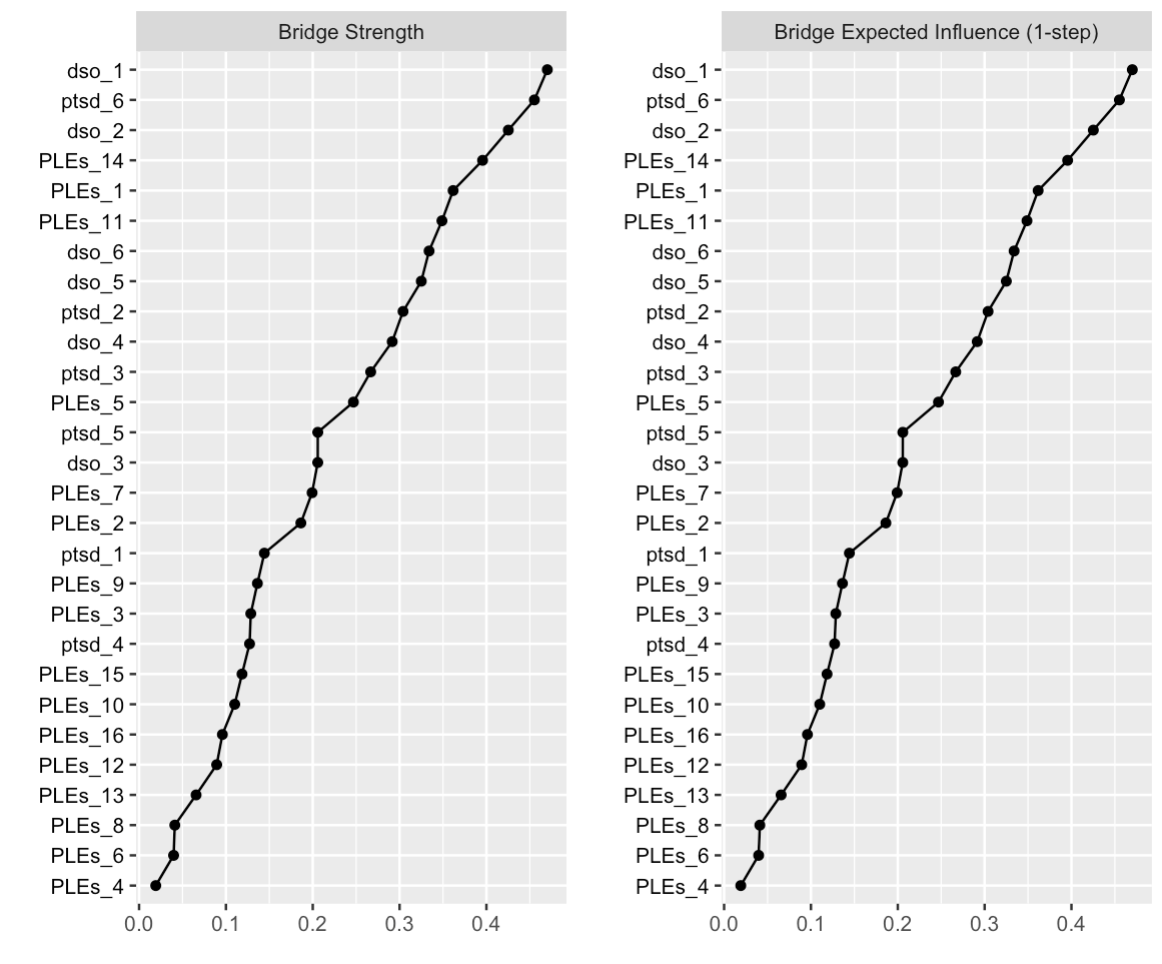
***

***Supplementary Figure 8 – Combination of Directed Acyclic Graphs for Male and Female Populations***

***
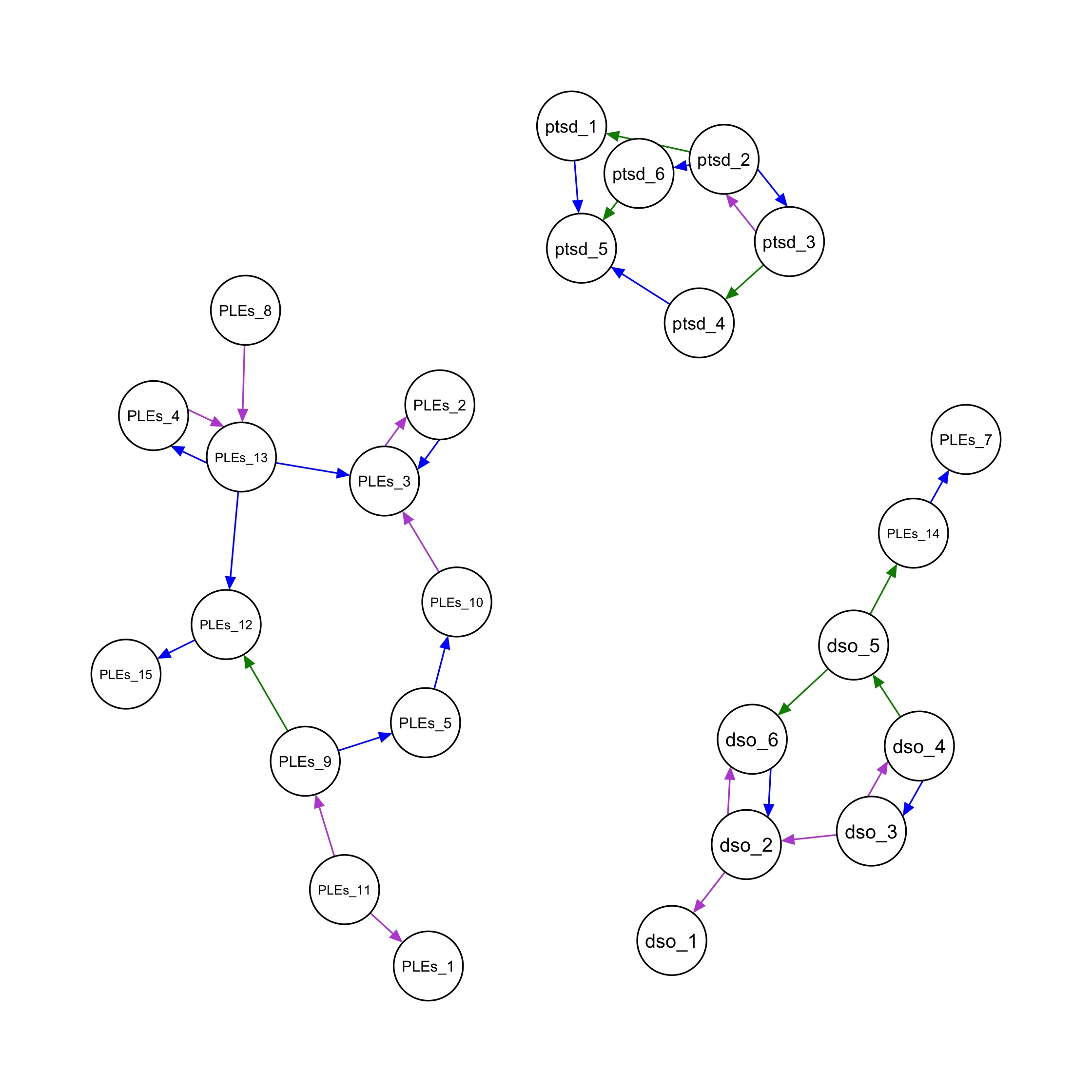
***

PTSD: Post-Traumatic Stress Disorder; DSO: Disturbances of Self-Organization; PLEs: Psychotic-Like Experiences. Violet arrows indicate a causal relationship in the female population; blue arrows indicate a causal relationship in the male population; green arrows indicate a causal relationship in both populations.

***Supplementary Table 1 – Explanation of Network’s Nodes***

| **Item Name** | **Explanation** |
| --- | --- |
| *ptsd_1* | Having upsetting dreams that replay part of the experience or are clearly related to the experience? |
| *ptsd_2* | Having powerful images or memories that sometimes come into your mind in which you feel the experience is happening again in the here and now? |
| *ptsd_3* | Avoiding internal reminders of the experience (for example, thoughts, feelings, or physical sensations)? |
| *ptsd_4* | Avoiding external reminders of the experience (for example, people, places, conversations, objects, activities, or situations)? |
| *ptsd_5* | Being “super-alert”, watchful, or on guard? |
| *ptsd_6* | Feeling jumpy or easily startled? |
| *dso_1* | When I am upset, it takes me a long time to calm down |
| *dso_2* | I feel numb or emotionally shut down |
| *dso_3* | I feel like a failure |
| *dso_4* | I feel worthless |
| *dso_5* | I feel distant or cut off from people |
| *dso_6* | I find it hard to stay emotionally close to people |
| *PLEs_1* | I feel uninterested in the things I used to enjoy |
| *PLEs_2* | I often seem to live through events exactly as they happened before (déjà vu) |
| *PLEs_3* | I sometimes smell or taste things that other people can’t smell or taste |
| *PLEs_4* | I often hear unusual sounds like banging, clicking, hissing, clapping or ringing in my ears |
| *PLEs_5* | I have been confused at times whether something I experienced was real or imaginary |
| *PLEs_6* | When I look at a person, or look at myself in a mirror, I have seen the face change right before my eyes |
| *PLEs_7* | I get extremely anxious when meeting people for the first time |
| *PLEs_8* | I have seen things that other people apparently can't see |
| *PLEs_9* | My thoughts are sometimes so strong that I can almost hear them |
| *PLEs_10* | I sometimes see special meanings in advertisements, shop windows, or in the way things are arranged around me |
| *PLEs_11* | Sometimes I have felt that I’m not in control of my own ideas or thoughts |
| *PLEs_12* | Sometimes I feel suddenly distracted by distant sounds that I am not normally aware of |
| *PLEs_13* | I have heard things other people can't hear like voices of people whispering or talking |
| *PLEs_14* | I often feel that others have it in for me |
| *PLEs_15* | I have had the sense that some person or force is around me, even though I could not see anyone |
| *PLEs_16* | I feel that parts of my body have changed in some way, or that parts of my body are working differently than before |

| **Variable** | **Median (IQR) / Numerosity (%)**  **Overall N = 972** | | **P-values** |
| --- | --- | --- | --- |
|  | Males  N = 492 | Females  N = 480 |  |
| *Age* | 18.58 (18.33-18.91) | 18.50 (18.33-18.83) | 0.2 |
| *Education*  Lyceum  Technical  Vocational | 189 (38%)  296 (60%)  7 (1.4%) | 313 (65%)  161 (34%)  6 (1.3%) | **<0.001** |
| *Trauma Diagnosis*  probable PTSD  probable cPTSD | 28 (5.7%)  10 (2.0%) | 61 (13%)  30 (6.3%) | **<0.001**  **<0.001** |
| *PLEs Diagnosis*  PLEs  no PLEs | 179 (36%)  313 (64%) | 233 (49%)  247 (51%) | **<0.001** |

***Supplementary Table 2 – Sample Characteristics as Broken Down by Gender***

IQR = Interquartile Range; PTSD = Post-Traumatic Stress Disorder; cPTSD = Complex PTSD; PLEs = Psychotic-Like Experiences;

***Supplementary Table 3 – Strength and Minimum Direction in the Whole Population***

| **Starting Node** | **Ending Node** | **Strength** | **Minimum Direction** |
| --- | --- | --- | --- |
| *ptsd_2* | *ptsd_1* | 1.0 | 0.6985 |
| *ptsd_2* | *ptsd_6* | 0.97 | 0.5283505 |
| *ptsd_2* | *PLEs_2* | 0.972 | 0.9331276 |
| *ptsd_3* | *ptsd_2* | 1.0 | 0.5525 |
| *ptsd_3* | *ptsd_4* | 1.0 | 0.5285 |
| *ptsd_4* | *ptsd_5* | 0.912 | 0.5926535 |
| *ptsd_6* | *ptsd_5* | 1.0 | 0.557 |
| *ptsd_6* | *dso_1* | 0.994 | 0.9044266 |
| *ptsd_6* | *PLEs_11* | 0.976 | 0.5594262 |
| *dso_2* | *dso_1* | 0.913 | 0.7102957 |
| *dso_3* | *dso_2* | 0.986 | 0.7991886 |
| *dso_4* | *dso_3* | 1.0 | 0.535 |
| *dso_4* | *dso_5* | 0.999 | 0.7397397 |
| *dso_5* | *dso_6* | 1.0 | 0.8265 |
| *dso_5* | *PLEs_14* | 0.999 | 0.8518519 |
| *dso_6* | *dso_2* | 1.0 | 0.5415 |
| *PLEs_3* | *PLEs_2* | 0.995 | 0.6221106 |
| *PLEs_3* | *PLEs_10* | 0.87 | 0.5275862 |
| *PLEs_4* | *PLEs_3* | 0.943 | 0.6611877 |
| *PLEs_5* | *PLEs_8* | 0.892 | 0.6093049 |
| *PLEs_5* | *PLEs_10* | 0.938 | 0.8486141 |
| *PLEs_9* | *PLEs_6* | 0.938 | 0.8560768 |
| *PLEs_9* | *PLEs_8* | 0.952 | 0.7447479 |
| *PLEs_9* | *PLEs_12* | 0.948 | 0.8765823 |
| *PLEs_10* | *PLEs_2* | 0.956 | 0.6291841 |
| *PLEs_11* | *dso_1* | 0.862 | 0.7981439 |
| *PLEs_11* | *PLEs_1* | 0.926 | 0.9200864 |
| *PLEs_11* | *PLEs_5* | 0.898 | 0.7884187 |
| *PLEs_11* | *PLEs_9* | 1.0 | 0.5755 |
| *PLEs_12* | *PLEs_3* | 0.882 | 0.6094104 |
| *PLEs_13* | *PLEs_3* | 0.954 | 0.7321803 |
| *PLEs_13* | *PLEs_4* | 0.985 | 0.5583756 |
| *PLEs_13* | *PLEs_8* | 0.981 | 0.5076453 |
| *PLEs_14* | *PLEs_7* | 0.951 | 0.9106204 |
| *PLEs_16* | *PLEs_15* | 0.927 | 0.5587918 |

**Supplementary Table 4 – Arc Strengths *in the Whole Population***

| **From** | **To** | **Strength** |
| --- | --- | --- |
| *ptsd_2* | *ptsd_1* | -160.318833 |
| *ptsd_2* | *ptsd_6* | -84.046852 |
| *ptsd_2* | *PLEs_2* | -25.751073 |
| *ptsd_3* | *ptsd_2* | -137.862707 |
| *ptsd_3* | *ptsd_4* | -307.827815 |
| *ptsd_4* | *ptsd_5* | -26.797066 |
| *ptsd_6* | *ptsd_5* | -119.950373 |
| *ptsd_6* | *dso_1* | -27.261485 |
| *ptsd_6* | *PLEs_11* | -74.425835 |
| *dso_2* | *dso_1* | -15.841914 |
| *dso_3* | *dso_2* | -46.995219 |
| *dso_4* | *dso_3* | -601.479871 |
| *dso_4* | *dso_5* | -231.712566 |
| *dso_5* | *dso_6* | -105.533272 |
| *dso_5* | *PLEs_14* | -102.367551 |
| *dso_6* | *dso_2* | -60.580828 |
| *PLEs_3* | *PLEs_2* | -14.809224 |
| *PLEs_3* | *PLEs_10* | -15.571813 |
| *PLEs_4* | *PLEs_3* | -14.934438 |
| *PLEs_5* | *PLEs_8* | -11.381579 |
| *PLEs_5* | *PLEs_10* | -24.754177 |
| *PLEs_9* | *PLEs_6* | -56.425624 |
| *PLEs_9* | *PLEs_8* | -15.030796 |
| *PLEs_9* | *PLEs_12* | -61.702974 |
| *PLEs_10* | *PLEs_2* | -9.971701 |
| *PLEs_11* | *dso_1* | -12.478156 |
| *PLEs_11* | *PLEs_1* | -48.958559 |
| *PLEs_11* | *PLEs_5* | -78.596902 |
| *PLEs_11* | *PLEs_9* | -125.044476 |
| *PLEs_12* | *PLEs_3* | -10.495955 |
| *PLEs_13* | *PLEs_3* | -15.97002 |
| *PLEs_13* | *PLEs_4* | -74.31451 |
| *PLEs_13* | *PLEs_8* | -32.236869 |
| *PLEs_14* | *PLEs_7* | -38.719418 |
| *PLEs_16* | *PLEs_15* | -33.333843 |

***Supplementary Table 5 – Strength and Minimum Direction in the Male Population***

| **From** | **To** | **Strength** | **Direction** |
| --- | --- | --- | --- |
| *ptsd_1* | *ptsd_5* | 0.917 | 0.5937841 |
| *ptsd_2* | *ptsd_1* | 1.0 | 0.5975 |
| *ptsd_2* | *ptsd_3* | 1.0 | 0.507 |
| *ptsd_2* | *ptsd_6* | 0.941 | 0.5504782 |
| *ptsd_3* | *ptsd_4* | 1.0 | 0.515 |
| *ptsd_4* | *ptsd_5* | 0.876 | 0.6238584 |
| *ptsd_6* | *ptsd_5* | 1.0 | 0.511 |
| *dso_4* | *dso_3* | 1.0 | 0.587 |
| *dso_4* | *dso_5* | 0.967 | 0.6277146 |
| *dso_5* | *dso_6* | 0.998 | 0.7249499 |
| *dso_5* | *PLEs_14* | 0.999 | 0.8503504 |
| *dso_6* | *dso_2* | 0.992 | 0.7056452 |
| *PLEs_2* | *PLEs_3* | 0.959 | 0.5067779 |
| *PLEs_5* | *PLEs_10* | 0.854 | 0.8079625 |
| *PLEs_9* | *PLEs_5* | 0.966 | 0.5802277 |
| *PLEs_12* | *PLEs_15* | 0.952 | 0.6018908 |
| *PLEs_13* | *PLEs_3* | 0.919 | 0.7317737 |
| *PLEs_13* | *PLEs_4* | 0.897 | 0.590301 |
| *PLEs_13* | *PLEs_12* | 0.926 | 0.7035637 |
| *PLEs_14* | *PLEs_7* | 0.884 | 0.7641403 |

***Supplementary Table 6 – Strength and Minimum Direction in the Female Population***

| **From** | **To** | **Strength** | **Direction** |
| --- | --- | --- | --- |
| *ptsd_2* | *ptsd_1* | 1.0 | 0.729 |
| *ptsd_3* | *ptsd_2* | 0.926 | 0.5858531 |
| *ptsd_3* | *ptsd_4* | 1.0 | 0.515 |
| *ptsd_6* | *ptsd_5* | 1.0 | 0.546 |
| *dso_2* | *dso_1* | 0.956 | 0.7751046 |
| *dso_2* | *dso_6* | 0.996 | 0.6129518 |
| *dso_3* | *dso_2* | 0.941 | 0.6705632 |
| *dso_3* | *dso_4* | 1.0 | 0.5085 |
| *dso_4* | *dso_5* | 1.0 | 0.752 |
| *dso_5* | *dso_6* | 0.971 | 0.6622039 |
| *dso_5* | *PLEs_14* | 0.932 | 0.8401288 |
| *PLEs_3* | *PLEs_2* | 0.86 | 0.7819767 |
| *PLEs_4* | *PLEs_13* | 0.903 | 0.5204873 |
| *PLEs_8* | *PLEs_13* | 0.975 | 0.5307692 |
| *PLEs_9* | *PLEs_12* | 0.979 | 0.8314607 |
| *PLEs_10* | *PLEs_3* | 0.853 | 0.6430246 |
| *PLEs_11* | *PLEs_1* | 0.944 | 0.8236229 |
| *PLEs_11* | *PLEs_9* | 1.0 | 0.5935 |

**Supplementary Table 7 – Arc Strengths in the Male Population**

| **From** | **To** | **Strength** |
| --- | --- | --- |
| *ptsd_1* | *ptsd_5* | -7.774821 |
| *ptsd_2* | *ptsd_1* | -87.185535 |
| *ptsd_2* | *ptsd_3* | -61.579812 |
| *ptsd_2* | *ptsd_6* | -25.958967 |
| *ptsd_3* | *ptsd_4* | -111.323018 |
| *ptsd_4* | *ptsd_5* | -7.62645 |
| *ptsd_6* | *ptsd_5* | -29.340639 |
| *dso_4* | *dso_3* | -264.971916 |
| *dso_4* | *dso_5* | -78.080509 |
| *dso_5* | *dso_6* | -58.694597 |
| *dso_5* | *PLEs_14* | -42.582154 |
| *dso_6* | *dso_2* | -45.203025 |
| *PLEs_2* | *PLEs_3* | -12.375849 |
| *PLEs_5* | *PLEs_10* | -16.629431 |
| *PLEs_9* | *PLEs_5* | -47.8477 |
| *PLEs_12* | *PLEs_15* | -22.848703 |
| *PLEs_13* | *PLEs_3* | -25.520395 |
| *PLEs_13* | *PLEs_4* | -35.758569 |
| *PLEs_13* | *PLEs_12* | -28.129479 |
| *PLEs_14* | *PLEs_7* | -13.883324 |

**Supplementary Table 8 – Arc Strengths in the Female Population**

| **From** | **To** | **Strength** |
| --- | --- | --- |
| *ptsd_2* | *ptsd_1* | -68.43673 |
| *ptsd_3* | *ptsd_2* | -65.25836 |
| *ptsd_3* | *ptsd_4* | -170.8976 |
| *ptsd_6* | *ptsd_5* | -100.51518 |
| *dso_2* | *dso_1* | -46.22999 |
| *dso_2* | *dso_6* | -33.02571 |
| *dso_3* | *dso_2* | -57.53321 |
| *dso_3* | *dso_4* | -319.89497 |
| *dso_4* | *dso_5* | -139.4808 |
| *dso_5* | *dso_6* | -16.87014 |
| *dso_5* | *PLEs_14* | -53.56039 |
| *PLEs_3* | *PLEs_2* | -16.7901 |
| *PLEs_4* | *PLEs_13* | -17.4682 |
| *PLEs_8* | *PLEs_13* | -27.91347 |
| *PLEs_9* | *PLEs_12* | -39.51695 |
| *PLEs_10* | *PLEs_3* | -18.79872 |
| *PLEs_11* | *PLEs_1* | -42.34201 |
| *PLEs_11* | *PLEs_9* | -79.08156 |
